# The Dorsal Medial Prefrontal Cortex Is Recruited by High Construal of Non-social Stimuli

**DOI:** 10.3389/fnbeh.2017.00044

**Published:** 2017-03-14

**Authors:** Kris L. M. R. Baetens, Ning Ma, Frank Van Overwalle

**Affiliations:** ^1^Experimental and Applied Psychology, Faculty of Psychology and Educational Sciences, Vrije Universiteit BrusselBrussels, Belgium; ^2^Center for Studies of Psychological Application, School of Psychology, South China Normal UniversityGuangzhou, China

**Keywords:** mentalizing, dmPFC, construal level, semantic retrieval, constraint

## Abstract

The dorsomedial prefrontal cortex (dmPFC) is part of the mentalizing network, a set of brain regions consistently engaged in inferring mental states. However, its precise function in this network remains unclear. It has recently been proposed that the dmPFC is involved in high-level abstract (i.e., categorical) identification or construction of both social and non-social stimuli, referred to as “high construal.” This was based on the observation of greater activation in the dmPFC shared by a high construal social condition (trait inference based on visually presented behavior) and a high construal non-social condition (categorization of visually presented objects) vs. matched low construal conditions (visual description of the same pictures). However, dmPFC activation has been related to task contexts requiring responses based on self-guided generation of mental content or decisions as compared to responses more directly determined by the experimental context (e.g., free vs. rule-governed choice). The previously reported dmPFC activity may reflect differences in task constraint (i.e., the extent to which the task context guided the process) confounded with the construal manipulation. Therefore, in the present study, we manipulated construal level and constraint independently, while participants underwent functional magnetic resonance imaging (fMRI). As before, participants visually described (low level construal) or categorized (high level construal) pictures of objects. Orthogonal to this, the description or categorization task had to be performed on either one object (low constraint) or on two objects simultaneously (high constraint), limiting the number of possible responses. Statistical analysis revealed common greater activation in both high construal conditions (high and low constraint) than in their low construal counterparts, replicating the influence of construal level on dmPFC activation (greater involvement in high than low construal), but no influence of constraint. In line with previous proposals and earlier work, we suggest that the dmPFC is involved in high-construal abstraction across different domains.

## Introduction

Inferring mental states of others consistently engages a number of brain areas, collectively called the mentalizing network (Frith and Frith, 2006; Van Overwalle and Baetens, 2009). Within this mentalizing network, the medial prefrontal cortex (mPFC) plays an essential role. While there is ample evidence that the ventral part of the mPFC subserves affective processes (for reviews, see e.g., Davidson and Irwin, 1999; Phan et al., 2002; Quirk and Beer, 2006; Roy et al., 2012), the dorsal part of the mPFC (dmPFC) appears to be involved in cognitively-oriented mentalizing, entailing reflective, and hypothetical social processing (see meta-analyses by Schilbach et al., 2012; Bzdok et al., 2013; Schurz et al., 2014; Molenberghs et al., 2016). Based on an extensive review, Van Overwalle (2011) concluded that the mPFC is preferentially engaged in social cognition: independent of the experimental task, activation of the mPFC is proportional to the amount of *mentalizing content* in the stimulus material (e.g., mental states such as goals of actions, beliefs, moral (in)justice, personality traits, social categories, and emotions). Nevertheless, several studies have reported activation in the dmPFC (centered around Montreal Neurological Institute coordinates 0, 50, 35 as specified by Van Overwalle and Baetens, 2009) without mentalizing content in the stimulus material (e.g., Goel et al., 1997; Ferstl and von Cramon, 2001, 2002; Grinband et al., 2006; Siebörger et al., 2007; Rowe et al., 2008; Green et al., 2010; Stern et al., 2010). Hence, it seems likely that the dmPFC subserves an underlying process that is required in, but not restricted to, social cognition. However, the precise nature of this fundamental process remains unclear. The present study investigates and compares two alternative explanations for this underlying process in dmPFC activation: (a) the level of abstract construal and (b) the lack of constraint.

Baetens et al. (2014) suggested that dmPFC involvement may be a function of the required construal level of the stimuli. According to construal level theory (CLT, Trope and Liberman, 2010), stimuli can be represented at different levels of abstraction or construal, with a higher construal level preserving certain central qualities (often conceptual, e.g., an aggressive trait) of stimuli (e.g., specific behaviors such as hitting, hurting, criticizing), while disregarding inessential, low-level features (often perceptual, e.g., whether physical force was used or not). This is not a process of mere information loss because high construal levels make the relations between stimuli more salient (e.g., very diverse behaviors can be classified as aggressive).

Using functional imaging, Baetens et al. (2014) found support for the association between high construal level and increased dmPFC activation. Importantly, they found stronger dmPFC activation not only for social stimuli as illustrated above, but also for non-social stimuli (e.g., means of transportation as a higher-level construal of a car, boat, or train). In their study, Baetens et al. (2014) presented pictures of an object or an actor engaged in everyday behaviors. In the high construal condition for persons, participants inferred traits from the depicted behavior. In the high construal condition for objects, they generated superordinate categories for visually presented objects (completions of the sentence “this is an example of …,” see Wakslak and Trope, 2009). For instance, by categorizing “a dog” as “an animal,” participants tend to focus on high-level superordinate aspects and disregard low-level attributes like tail, fur, etcetera. In low construal conditions regarding both persons and objects, participants generated visual descriptions regarding color, shape, structure, and texture of the same pictures. The dmPFC and other parts of the mentalizing network were significantly more active in the high than in the low construal tasks, both for object and person pictures.

This result raises the question why and to what extent high construal instructions induce dmPFC activation across a variety of domains. One possibility is that the dmPFC is predominantly involved in social processes, such as judgments about the self and others (Northoff et al., 2006; Denny et al., 2012). These judgments typically entail high construals: abstract qualities like preferences, traits, or opinions of the actor, which are mental rather than directly perceivable. The social brain hypothesis (Dunbar, 1998, 2003) suggests that this social abstraction function developed early during human evolution due to greater demands enforced by living in increasingly large social groups that facilitate cooperation and hence survival. Subsequently, this abstraction function may have been re-used and extended to facilitate categorization of an increasing variety and complexity of other objects, including objects from human (e.g., tools) or biological nature (e.g., food) which are essential in production and foraging. Indeed, a direct comparison between social and non-social high construal judgments in Baetens et al. (2014) revealed a stronger activation of the dmPFC in social high construals. Likewise, Moran et al. (2011) found greater dmPFC activation in judgments of character (social) vs. appearance (non-social) of self and others. Obviously, this explanation predicts dmPFC involvement in abstract tasks without mentalizing content as well.

While the findings of the study by Baetens et al. (2014) are consistent with this account, it is possible that the higher dmPFC activation in high vs. low construal is actually driven by differences in task constraint (i.e., the extent to which the task context guided the cognitive process). Prior research documented stronger dmPFC activation in the comparison of free vs. rule-governed choice (Brass and Haggard, 2007; Rowe et al., 2008), during mind-wandering (Mason et al., 2007b) and internally guided or self-generated thought (Andrews-Hanna et al., 2014). For example, in a study on free vs. rule governed choice (Rowe et al., 2008), the comparison of free choice (e.g., freely select one of four buttons) with rule-governed choice (e.g., press button designated by arrow) yielded activation of the dmPFC. In a subsequent experiment, prior to the presentation of a stimulus array, participants were prompted either to apply a forced rule to the subsequent display (e.g., indicate the location of the visually darkest stimulus) or to freely choose between two rules (e.g., choose to either indicate the location of the darkest or the lightest stimulus in the subsequent presentation). Again, dmPFC activation was associated with the unconstrained process of voluntary rule selection as opposed to the more constrained process of rule application. One could argue that in the study by Baetens et al. (2014), the visual (low construal) task was much more constrained than the categorization (high construal) task. That is, in the visual task, participants could employ a systematic guiding strategy to describe the pictures (e.g., scan visually and describe color, shape, texture, structure), while such constraining strategy was unavailable in the categorization task of the pictures.

**Figure 1:**
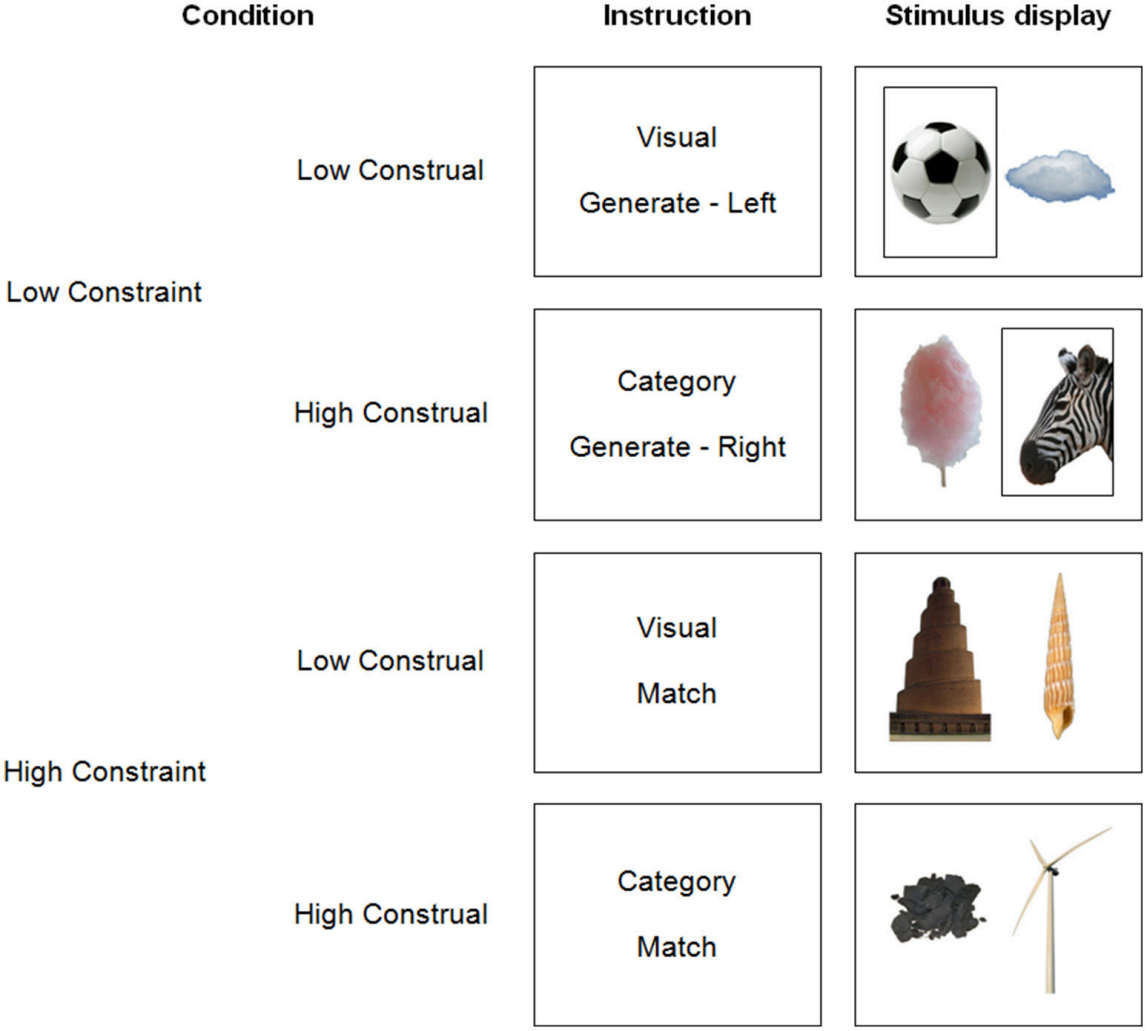
**Illustration of the four trial types**.

In the present study, we tried to replicate the impact of construal level on dmPFC activation, while investigating the potential influence of constraint. To this end, we manipulated construal level and constraint independently within one experiment. The design (Figure 1) was an extension of Baetens et al. (2014). First, in a replication of this prior study, we manipulated construal level by asking participants to either categorize (high construal) or visually describe (low construal) pictures of objects (manmade objects, animals, and natural phenomena). As noted earlier, categorizations (i.e., finding completions for the sentence “This is an example of…”) prompt higher construals of stimuli than visual descriptions (Wakslak and Trope, 2009). Second, as a novel manipulation, we implemented a low vs. high constraint level of the task. However, a straightforward application or extension of earlier constraint manipulations is impossible, because we request in all conditions to follow some rule to manipulate construal level (i.e., visual vs. categorical judgment). Therefore, we decided to render the application of this rule itself more or less constrained. In low constraint trials, we asked participants to freely *generate* categories or visual characteristics pertaining to only one picture. In contrast, in high constraint trials, they performed the same task for two pictures at once, with the constraint that their categorizations or visual descriptions had to *match* between the two pictures. Thus, the number of possible answers was strictly smaller in high than in low constraint trials. Further, the number of visual or conceptual correspondences between the stimuli was limited, while one commonality was highlighted, reducing the search space. Note that this implementation of constraint is more subtle than in prior research, and might therefore potentially remain undetected.

If dmPFC activity is triggered by a greater required construal level (i.e., abstraction), this should lead to stronger dmPFC involvement in high construal (category) vs. low construal (visual), regardless of constraint. Alternatively, if dmPFC activation is driven by self-guided, unconstrained processes, we would expect stronger involvement in low (generate) than in high (match) constraint conditions, regardless of construal level.

## Materials and methods

### Participants

All participants had normal or corrected-to-normal vision and a normal neurological history.

Ten women and nine men, right-handed, with ages between 19 and 51 (*M* = 26) participated in exchange for €10. One other participant was excluded due to excessive movement artifacts (detailed below). This study was carried out in accordance with the recommendations of Medical Ethics Committee at the Ghent University Hospital and the Brussels University Hospital with written informed consent from all subjects. All subjects gave written informed consent in accordance with the Declaration of Helsinki. The protocol was approved by the Medical Ethics Committees at the Ghent University Hospital and the Brussels University Hospital.

### Stimulus material

The pictures depicted everyday manmade objects or structures (e.g., a chair), non-manmade objects, such as animals (e.g., a cow), plants, or parts thereof (e.g., a leaf), and natural phenomena or structures (e.g., a mountain), all on a white background. The whole stimulus set consisted of 120 images. All images were cropped to 348 × 522 pixels and presented side by side. Figure 1 shows example pictures.

The images consisted of 60 pairs of pictures. Perceptual pairs (*n* = 30) shared at least one visual characteristic: color or color pattern (e.g., black-and-white), shape or structure (e.g., round), or texture (e.g., fluffy). Conceptual pairs (*n* = 30) shared at least one common semantic category. In the high constraint conditions, conceptual or perceptual pairs were presented together as just described. In the low constraint conditions, we presented pseudo-random combinations of the images, as detailed in the experimental procedure below.

### Experimental procedure

**Figure 2:**
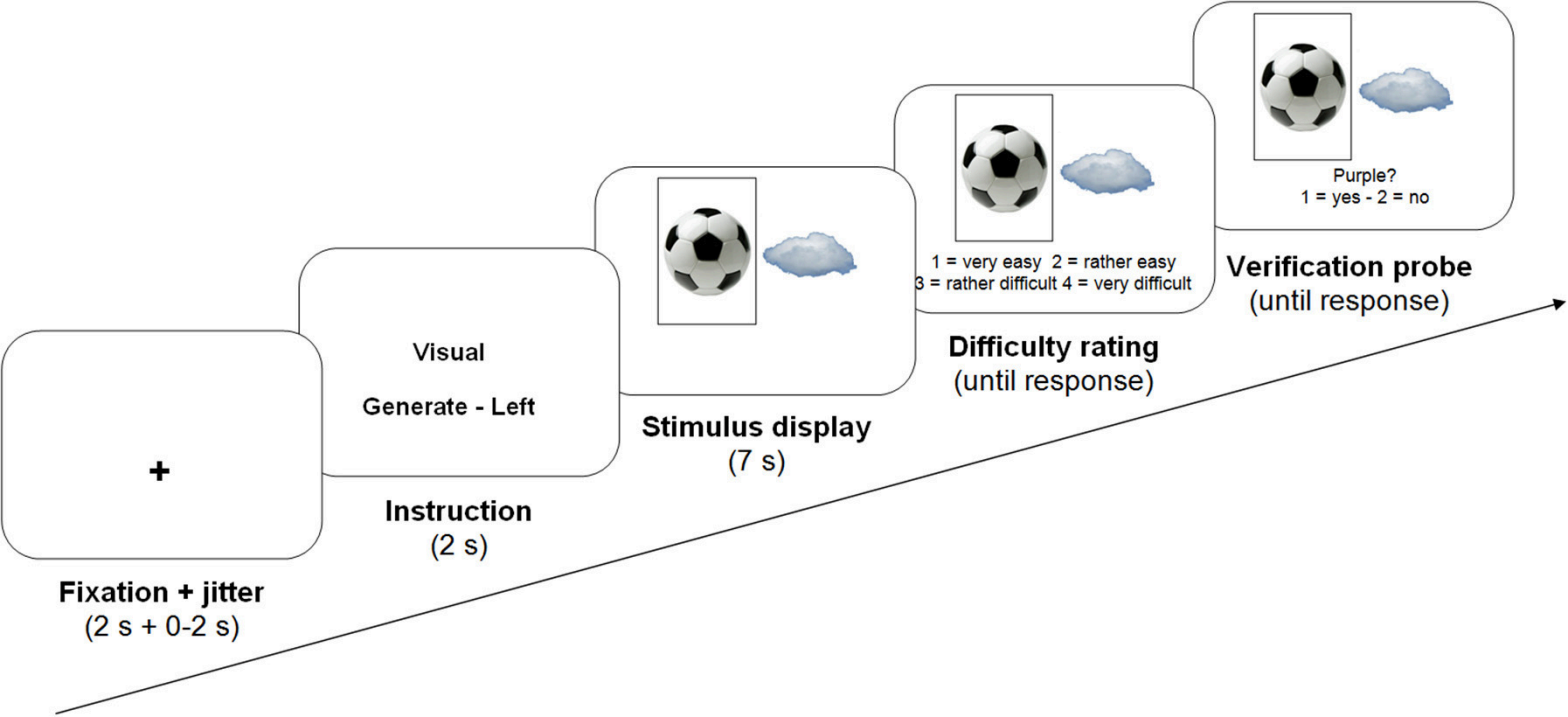
**Structure of an example trial**.

Each trial consisted of a fixation cross (2 s), variable 0–2 s of jitter, an instruction (2 s), two pictures depicting one object each (7 s), a difficulty rating question and finally a verification probe, both of which remained on screen until participants responded (see Figure 2 for an overview of the presentation procedure). The instructions informed participants whether they were to categorize or describe the pictures during the next trial (construal level manipulation), and whether they had to take only one or both pictures into account simultaneously (constraint manipulation). In the latter case, they were informed whether they had to perform the task on the left or the right image (50% each, randomly determined).

We manipulated construal level independently from constraint, resulting in 4 trial types. In the low construal/high constraint trials, we presented perceptual pairs. Participants generated as many as possible visual characteristics (color, texture, shape, or structure) that applied to both presented objects. Participants were explicitly asked not to generate subjective or evaluative descriptions or interpretations. In the high construal/high constraint trials, we presented conceptual pairs, and participants generated as many categories as possible to which both depicted objects belonged. As an aid, they were told to generate sentence completions of “These are an example of…,” in line with Wakslak and Trope's (2009) manipulation to prime a high construal mindset. They were told that subcategories of previously generated categories (e.g., “mammal” after “animal”), were considered valid, but categories based on visual features (e.g., “yellow things”) or on subjective grounds (e.g., “ugly things”) were not.

In the low constraint conditions, there were also two pictures on screen to keep the visual input equivalent. Participants performed the same task, but were instructed to take only the picture on the left or on the right of the screen into consideration, as indicated in the instructions and by a black frame around the relevant picture during stimulus presentation (see Figure 1). In the low construal/low constraint trials, participants freely generated as many visual characteristics of the target object as possible. In the high construal/low constraint trials, participants generated categories for the single target object. To avoid participants performing the same judgment (describing or categorizing) on the same picture twice in the course of the experiment, the target picture in the low construal/low constraint condition was randomly selected from the conceptual pairs in the high construal/high constraint condition. The target picture in high construal/low constraint condition was selected from the perceptual pairs. Non-target pictures were randomly selected from the remaining images.

In all conditions, participants were requested to generate responses during the entire 7 s stimulus presentation. There were 30 trials per condition, all presented in a fully random order. After stimulus presentation, each trial, participants rated task difficulty on a four-point scale (1 = very easy, 2 = rather easy, 3 = rather difficult, 4 = very difficult), by pressing one of four buttons on a response box below their left hand. There was no time limit for responding to this question. Following this difficulty judgment, participants were presented a verification probe, a category or visual feature that was clearly applicable or inapplicable to the picture(s) that had just been presented (50% each, in every condition), as determined at face value. Participants indicated whether they had thought of this category or feature during the trial, providing a behavioral performance measure.

Participants performed 10 practice trials inside the scanner before the start of the experiment to allow them to get used to the experimental environment.

### Practice procedure

Prior to the experiment in the scanner, participants performed the experimental tasks out loud in the presence of the first author. They received feedback on how well they followed the instructions. When participants performed all the tasks correctly, they were asked whether they felt confident about their performance. When they expressed confidence, we proceeded to the actual experiment. Otherwise, additional training trials were provided. Participants were informed that the tasks in the scanner were identical, save that they were to be performed silently, as to avoid motion artifacts.

### Imaging procedure

We collected images with a 3 T Magnetom Trio MRI scanner system (Siemens Medical Systems, Erlangen, Germany), and an 8-channel radiofrequency head coil. Participants could see the monitor display projected on a screen at the end of the scanner bore by means of a mirror on the head coil. For stimulus presentation, we used E-Prime 2.0 software (www.pstnet.com/eprime; Psychology Software Tools). In order to provide comfort and minimize movement artifacts, we placed foam cushions inside the head coil. After acquisition of a high resolution T1-weighted structural scan (MP-RAGE), there was one functional run featuring a gradient-echo echoplanar pulse sequence (EPIs; 30 axial slices; 4 mm thick; 1 mm skip; TR = 2 s; TE = 33 ms; 3.5 × 3.5 × 4.0 mm in-plane resolution).

### Image processing and statistical analysis

The fMRI data were preprocessed and analyzed with SPM8 (Wellcome Department of Cognitive Neurology, London). Preprocessing involved (1) slice timing correction, (2) realignment to correct for head movement, (3) coregistration of the participant's high resolution anatomical data to their mean EPI, (4) normalization into standard MNI space (rescaled to 2 × 2 × 2 mm isotropic voxels) based on the ICBM 152 brain template (Montreal Neurological Institute), and (5) spatial smoothing (6 × 6 × 6 mm full-width at half maximum Gaussian kernel).

Next, we used the Artifact Detection Tool software package (ART; http://web.mit.edu/swg/art/art.pdf; http://www.nitrc.org/projects/artifact_detect) to remove excessive motion artifacts and control for correlations between movement parameters or global mean signal and the experimental design. To detect movement outliers, between-scan differences in a temporal difference series were assessed using a Z-threshold of 3, a scan to scan movement threshold of 0.45 mm and a rotation threshold of 0.02 rad. Based on this analysis, one participant was excluded from further analysis, resulting in a final total of 9 male and 10 female participants. There were no problematic correlations between movement parameters or global mean signal and the experimental design.

The statistical analysis entailed two levels. First, we estimated the single-subject effects using a general linear model, using a canonical response function and a 128 s high-pass filter. The first level analysis modeled the effects of the four conditions of interest, time-locked at the presentation onset of each picture pair, and using an event duration of 7 s (the time allotted to generate responses). Identical analyses using an event duration of 0 s focusing on the onset of the stimulus presentation are reported in the Supplementary Materials (Table S1). Further, the first level model included several nuisance regressors: 6 motion parameter estimations from the realignment procedure as well as an additional regressor for each movement outlier (as identified by ART). Serial correlations were accounted for by the default auto-regressive AR(1) model.

To investigate group-level effects, we conducted a repeated measures analysis of variance (ANOVA) on the parameter estimates associated with each trial type, with Constraint (generate vs. match) and Construal Level (visual vs. category) as within-participants factors, including a participants factor to account for between-participant variance. To account for variance explained by differences in difficulty between conditions, we included a covariate with the mean difficulty rating per participant per condition (overall mean centered, no interactions specified). Comparisons of interest were implemented as linear contrasts using a random-effects model. As a main effect of either Construal Level or Constraint could be due to one of both individual conditions that made up each factor (e.g., effects of Construal level might be observed under low or high constraint, or both), we conducted conjunction analyses (conjunction null) to identify regions more active in high than low construal, regardless of constraint and vice versa. An uncorrected threshold of *p* ≤ 0.001 and a minimum cluster size of 10 rescaled (2 × 2 × 2 mm) voxels were used for this whole-brain analysis, followed up by a peak level FWE-corrected threshold of *p* ≤ 0.05.

Lastly, to identify brain regions showing activation as a linear function of the difficulty rating across conditions, we conducted a separate two-level analysis. At the single-subject level, all trials were modeled as belonging to one and the same condition, with the difficulty rating for each trial as a parametric modulator. We tested for group-level effects by means of one sample *t*-tests (testing for significantly positive and negative slopes of the parametric modulator), treating participant as a random effect and using the same statistical thresholds as described above.

### Analysis of behavioral data

The statistical analysis of the behavioral measures (difficulty ratings and probe acceptance data) involved repeated measures ANOVAs with the same within-participant factors as the whole-brain imaging analysis.

## Results

### Behavioral measures

Table 1 provides descriptive statistics of the performance measures. A repeated measures ANOVA on the difficulty ratings with Constraint and Construal Level as within-participant factors revealed a significant main effect of Constraint, *F*_(1, 18)_ = 12.75, *p* < 0.01 and a significant interaction between Constraint and Construal Level, *F*_(1, 18)_ = 7.49, *p* < 0.05. *Post-hoc* paired samples *t*-tests indicated that the difficulty ratings in both high constraint conditions were significantly higher than in their low constraint counterparts (*p*s < 0.05).

**Table 1:** **Behavioral data per condition (***N*** = 19)**.

	**Low constraint**		**High constraint**	
	**Low construal**	**High construal**	**Low construal**	**High construal**
Difficulty rating: Mean (*SD*)	1.86 (0.29)	2.05 (0.35)	2.16 (0.39)	2.18 (0.39)
Implausible probes accepted (*SD*)	4% (7%)	0% (2%)	4% (7%)	1% (3%)
Plausible probes accepted (*SD*)	54% (14%)	66% (17%)	84% (12%)	85% (8%)

**Figure 3:**
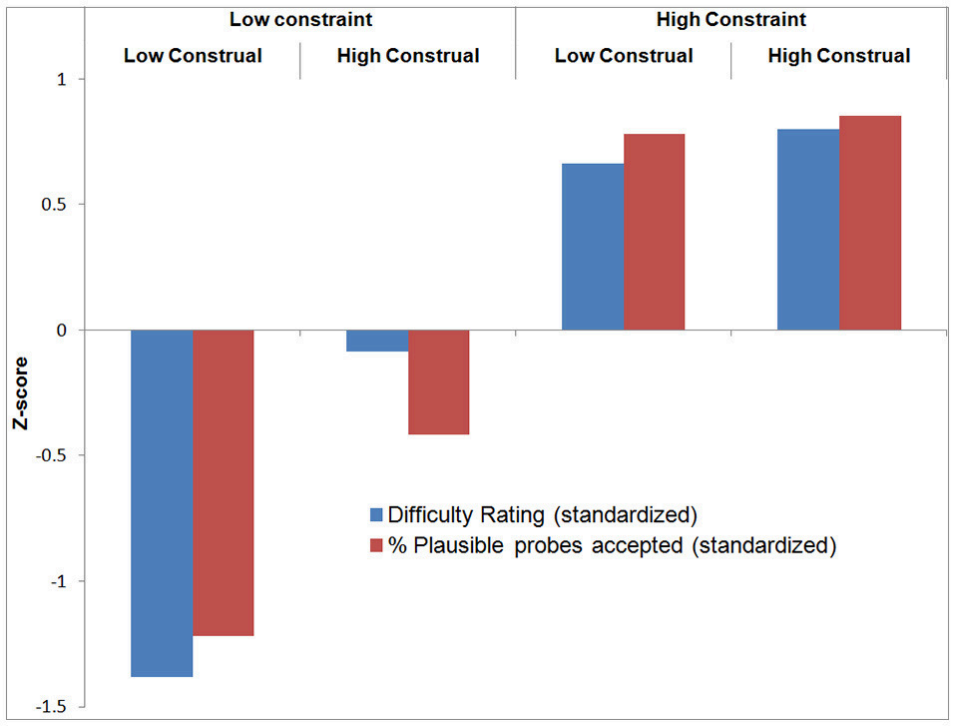
**Relationship between difficulty rating and plausible probe acceptance**.

With respect to the verification probes, recall that these queried whether participants had thought of an (experimenter-provided) plausible or implausible category or feature during the trial. Participants rejected *implausible* probes in on average 98% of the trials, suggesting that they performed the task well (Table 1). A repeated measures ANOVA revealed no significant differences between conditions. A repeated measures ANOVA on the proportion of accepted *plausible* probes per condition revealed a significant main effect of Constraint [*F*_(1, 18)_ = 73.78, *p* < 0.001], Construal Level [*F*_(1, 18)_ = 7.33, *p* < 0.05], and their interaction [*F*_(1, 18)_ = 4.78, *p* < 0.05]. Overall, this pattern is quite similar to that of the difficulty ratings. To enable direct visual comparison between the difficulty ratings and plausible probe acceptance, we standardized these variables by subtracting the mean and dividing by the standard deviation across conditions per participant. The results are displayed in Figure 3 (all statistical analyses were carried out on raw data).

As a manipulation check to verify that participants followed the instructions, we computed two correlations. First, comparing *between participants*, we found a *negative* correlation between overall mean task difficulty (across conditions) and mean plausible probe acceptance (across conditions) per participant (*r* = −0.56, *p* < 0.05). This provides convergent validity for these measures: participants who found the task difficult, considered, and generated plausible probes less often and vice versa. On the other hand, comparing *between conditions* (see Figure 3), we found a significant *positive* correlation between difficulty rating and plausible probe acceptance (mean across participants *r* = 0.30, *p* < 0.05). The more difficult a condition was judged, the more frequently participants had generated the experimenter-provided plausible probes in these conditions. Most likely, this is because there were, as intended, less plausible answers in these difficult (i.e., high constraint) conditions.

### fMRI data

The whole-brain analysis revealed a main effect of Construal on dmPFC activation, while there was no such main effect of Constraint, nor an interaction with Construal Level. Because taking up task difficulty as a covariate of no interest could bias the results toward or against one of the hypotheses regarding the involvement of the dmPFC, we replicated the analysis without this covariate. The results regarding the dmPFC were identical: only in the comparison of high vs. low construal did we find significant activation in this region.

**Figure 4:**
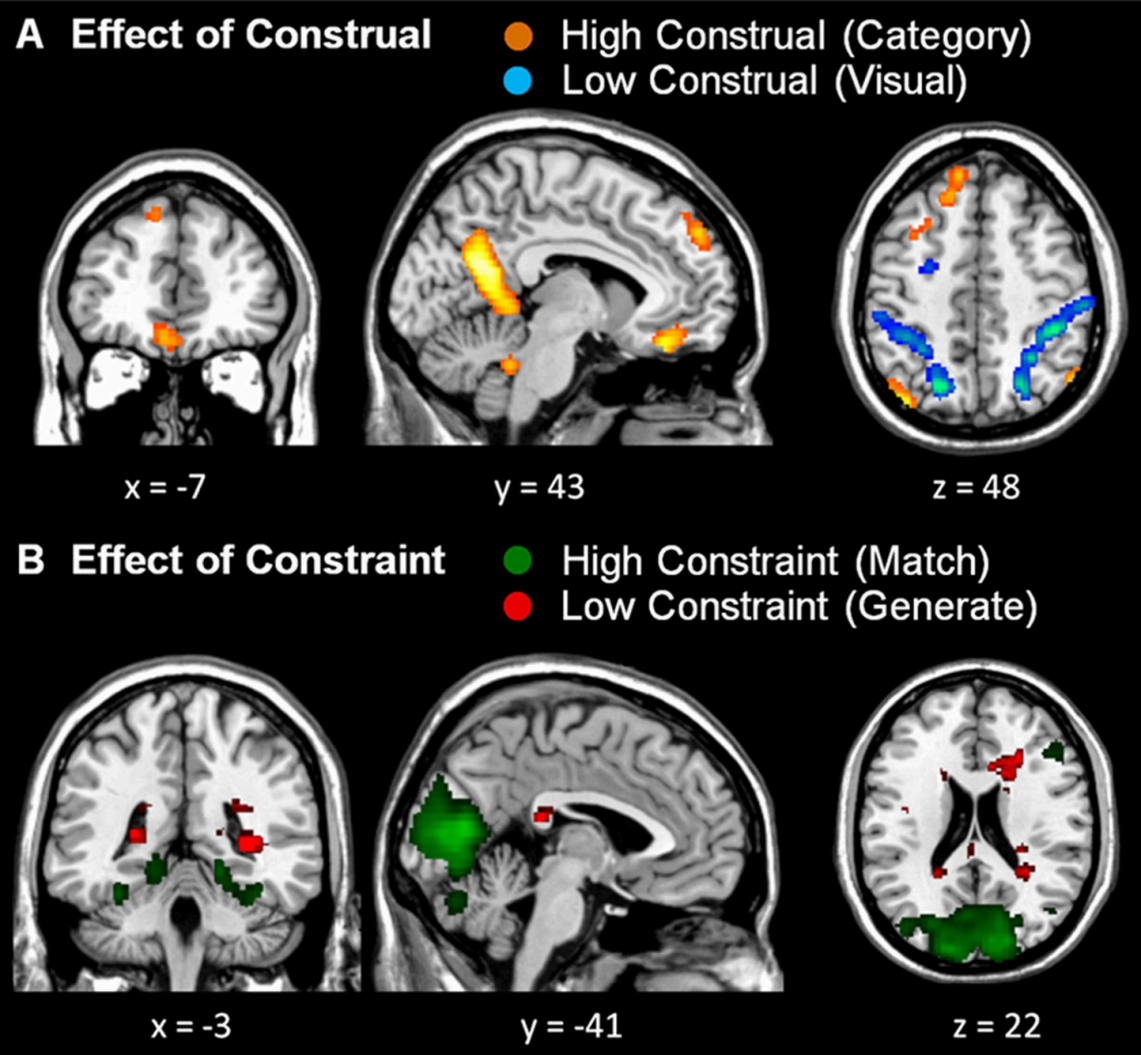
**Conjunction analyses. (A)** Conjunction of both high construal level > both low construal level conditions (orange) and both low construal level > both high construal level conditions (blue). **(B)** Conjunction of both high constraint > both low constraint conditions (green) and both low constraint > both high constraint conditions (red). All uncorrected whole-brain threshold of *p* < 0.001, number of voxels > 10; trial duration 7 s and difficulty included as a covariate of no interest.

Although, there was no significant interaction in dmPFC activation, to ensure that the significant main effect of Construal was not driven by one of the (high or low) constraint conditions, we computed conjunction analyses of the high vs. low construal conditions across the Constraint factor, and likewise of the high vs. low constraint conditions across the Construal factor (see Table 2 and Figure 4). Under these more stringent conditions, too, we found significantly stronger activation in the dmPFC in high vs. low construal level conditions, as well as in the ventromedial PFC, middle temporal gyrus, parahippocampal gyrus, precuneus angular gyrus, inferior parietal lobule, and cerebellum.

**Table 2:** **Results of conjunction analyses (vs. null), all at threshold ***p*** < 0.05 (FWE-corrected, number of voxels ≥ 10), trial duration 7 s**.

**Contrast**	**Anatomical region**	**MNI coordinates**					
		**BA**	***x***	***y***	***z***	***t***	***k***
High construal > Low construal *(for both levels of constraint)*	Dorsomedial prefrontal cortex	9	−8	52	38	4.85	434
	Ventromedial prefrontal cortex	11	−6	36	−20	5.35	246
	*Superior frontal gyrus*	*8*	−*30*	*16*	*56*	*5.48*	*860*
	Middle temporal gyrus	21	−48	−12	−22	5.33	419
	Parahippocampal gyrus	30	−14	−46	4	6.02^*^	1,722^a^
	Precuneus (inferior)	31	−10	−60	22	6.7^*^	1,722^a^
	Angular gyrus	39	−42	−64	30	5.79^*^	782^b^
		39	54	−66	34	6.12^*^	318^c^
		39	52	−66	42	5.81^*^	318^c^
		39	−48	−72	38	6.79^**^	782^b^
	Inferior parietal lobule	7	−42	−74	46	6.10^*^	782^b^
	Cerebellum		16	−86	−34	5.79^*^	394
Low construal > High construal *(for both levels of constraint)*	Inferior frontal gyrus	9	52	10	32	7.04^**^	921^d^
		44	50	10	24	6.67^**^	921^d^
		9	−46	6	30	8.56^**^	1,889^e^
	Superior frontal gyrus	6	−24	0	62	8.46^**^	1,889^e^
		6	26	0	58	7.61^**^	778
	Middle frontal gyrus	6	−24	−4	52	8.03^**^	1,889^e^
	Superior parietal lobule	7	−24	−60	58	9.76^**^	9,055^f^
		7	26	−68	42	10.42^**^	9,100^g^
	Precuneus (superior)	7	24	−60	54	10.29^**^	9,100^g^
		7	−24	−68	38	11.47^**^	9,055^f^
	**Precuneus (superior)**	7	−20	−68	50	10.18^**^	9,055^f^
	Inferior temporal gyrus	37	54	−62	−8	10.24^**^	100
	Cerebellum		14	−70	−44	5.55	44
	*Inferior parietal lobule*	*40*	−*34*	−*46*	*54*	10.01^**^	
	*Middle temporal gyrus*	*37*	−*46*	−*64*	*2*	10.22^**^	
Low constraint > High constraint *(for both levels of construal)*	No significant clusters						
High constraint > Low constraint *(for both levels of construal)*	Lingual gyrus	18	−10	−76	−2	13.05^**^	15,838^h^
	Cuneus	17	12	−80	6	12.05^**^	15,838^h^
		18	−8	−88	10	11.67^**^	15,838^h^
	*Middle frontal gyrus*	*9*	*48*	*26*	*38*	*5.64*	
		*46*	*48*	*32*	*28*	*5.60*	

*BA, Brodmann's Area; L and R, left and right hemispheres; t, t-score at those coordinates (peak value); k, cluster size (in voxels). Regions with ks that share a superscript originate from the same cluster. Activations in bold are only significant in the model including difficulty as a covariate of no interest; activations in italics are only significant without including difficulty as a covariate of no interest*.

* *p < 0.01*;

** *p < 0.001 (FWE-corrected)*.

Like in the study by Baetens et al. (2014), the reported dmPFC activation was rather left of the midline. The area typically involved in mentalizing tasks according to Van Overwalle and Baetens' meta-analysis (2009) is centered at the midline, around MNI coordinates 0, 50, 35. To explore whether this region was also more active in the high than low construal level conditions, within the conjunction contrast we performed a small volume correction analysis within a 6 mm sphere around these coordinates. This yielded significant activation (MNI −4, 52, 38, *t* = 4.26, *p* < 0.01, FWE-corrected), confirming that the mentalizing region in the dmPFC was also more involved in the high than low construal level conditions in the present study.

The whole-brain conjunction analysis further revealed that low construal was associated with more prominent activations in the lateral frontal and parietal cortex, as well as the inferior temporal cortex (Table 2). With respect to constraint, high constraint was associated with activation in the visual cortex, regardless the level of construal. No activations survived the statistical threshold for low constraint.

Finally, to verify whether dmPFC activation was driven by task difficulty, we identified brain regions in which activation was a linear function of the difficulty rating over all experimental conditions. This parametric analysis yielded no peaks surviving the statistical threshold, and neither at a more lenient FDR-corrected threshold. We performed a similar small volume correction analysis within a 6 mm sphere around the dmPFC, and again found no significant activations.

## Discussion

Previous research has demonstrated dmPFC involvement in high vs. low construal of both social and non-social stimuli (Baetens et al., 2014). In this study, for non-social material, we explored the possibility that this activation is modulated by the degree of task constraint. However, we found no effect of constraint on dmPFC activation, nor any significant interaction of this factor with construal level. Instead, we found greater involvement of the dmPFC given high as opposed to low construal, irrespective of constraint, and subjective task difficulty. This result confirms that higher construal recruits the dmPFC as revealed in prior work by Baetens et al. (2014).

The proposal that the dmPFC subserves high construal is consistent with its prominent role in abstract, social, and affective processes which require the computation of mental states. The crucial role of the dmPFC in mentalizing (Van Overwalle and Baetens, 2009) and self or other-related judgments (Northoff et al., 2006; Van Overwalle, 2009; Denny et al., 2012) is understandable from this point of view, as person judgment tasks typically feature relatively abstract (high construal level) qualities. This is in line with an important distinction within this domain, namely the relatively higher dmPFC involvement in enduring (e.g., trait) than in temporary (e.g., goal) person inferences (Van Overwalle and Baetens, 2009) or in character than in appearance judgments (Moran et al., 2011).

### Explaining the role of the dmPFC in high construal

The finding of this study that high construal was observed for non-social stimuli suggests that the dmPFC may subserve a more general, underlying cognitive function that is particularly important for social cognition as repeatedly demonstrated in earlier research (Van Overwalle, 2009; Schurz et al., 2014), but also potentially valuable in other contexts. This is consistent with a related position recently put forward by Spunt and Adolphs (2014), who argued that high construal might consist of a set of processes that are part of our abilities to think about the internal state of other people. This modular view allows for the possibility that one of these processes computes high-construal categories that are not observable but only exist in one's mind. This functional module may be profitable applied in other non-social contexts for constructing and understanding non-observable high-construals. Thus, unlike cars and boats, transportation means as such cannot be observed in reality or imagined vividly, but might nevertheless be constructed internally on logical grounds (e.g., based a shared function).

This modular position is consistent with an evolutionary view on social cognition mentioned earlier, which posits that the brain's expansion during human evolution was partly driven by increasing pressure to maintain life-long relationships with large numbers of conspecifics, requiring abstract judgments on their qualities and traits to work and live together. Extending this view, this abstraction function might have been re-used for judging abstract qualities of non-social manmade (i.e., tools) or natural objects (i.e., food), and are crucial for (communication about) production and nutrition so that their abstract categorization becomes evolutionary advantageous. This evolutionary trajectory is supported by research demonstrating stronger dmPFC involvement in social as opposed to non-social high construals (Baetens et al., 2014).

### Alternative interpretations

Nevertheless, several alternative accounts could explain the pattern of dmPFC activation in the present study. One possible explanation is that high construal relies on domain-general semantic processes. Specifically, the dmPFC might be “responsible for the context-dependent representation of, and choice between, alternative lexical semantic interpretations” and so contribute to a general executive system that “uses context to relate external stimuli to specific internal representations of knowledge about these stimuli” (Scott et al., 2003, p. 874). Note that the reference to “internal representations” has some similarity with the modular view of internal processes during mentalizing mentioned earlier. A meta-analysis of over 120 studies by Binder et al. (2009), showed that the left dmPFC is consistently more activated by processing the meaning of words vs. their structural properties (i.e., phonology and orthography). As high construals require disregard of low-level perceptual features in favor of central qualities, there is a strong analogy between high vs. low construal level and the processing of meaning vs. structural properties of words. Other research demonstrates that the dmPFC is involved in the creation of new metaphors, arguably the pinnacles of abstract language use (Benedek et al., 2014) and in coherence building during discourse comprehension (Ferstl and von Cramon, 2001, 2002; Siebörger et al., 2007; Ferstl et al., 2008), in which retrieval of the proper level of abstraction is crucial for achieving coherence (Ferstl and von Cramon, 2002). In conclusion, this alternative account suggests that dmPFC activation in high construal could be driven not by high construal itself, but by a demand on semantic retrieval processes (i.e., choice between context-dependent interpretations). It also dovetails nicely with greater dmPFC involvement in improvising vs. reproducing of hip hop lyrics (Liu et al., 2012) but not of an instrumental jazz solo (Limb and Braun, 2008).

A second alternative explanation, also in line with a modular account, views activation in cortical midline structures, and the dmPFC in particular, as associated with internally focused processes, as opposed to externally focused processes which are associated with lateral (prefrontal) cortex activation (for a review, see Lieberman, 2007; for a discussion regarding the difference between constraint and internally oriented processes, see Mason et al., 2007a). This distinction could explain dmPFC involvement in the high construal conditions, because as construal level increases, specific stimulus characteristics (externally, perceptually available information) become less important compared to relations with other stimuli (internally represented information). However, experimental evidence argues against the view that the internal-external orientation *per se* is related to dmPFC activation in mentalizing, or more generally in high construal. Several studies actually suggest the inverse pattern: greater involvement of the medial dmPFC in externally focused processes and of the lateral prefrontal cortex in internally focused processes (Burgess et al., 2007; Henseler et al., 2011). Critically, Gilbert et al. (2007) made a within-participants comparison of mentalizing (performing a task supposedly with a collaborator) vs. no mentalizing (no collaborator) on the one hand, and externally vs. internally oriented processes on the other, and found activations in dissociable parts of the dmPFC related to mentalizing and internal orientation.

The present results are at odds with a previous study by Gilead et al. (2014). Like Baetens et al. (2014), these authors attempted to isolate regions involved in high vs. low construal level of persons and objects. Two key findings emerged from the Gilead et al. (2014) study. First, there was no significant activation in the contrast of high vs. low construal level. This is inconsistent with previous studies consistently revealing higher dmPFC activation in the why vs. how contrast, using both verbal and visual stimuli (Spunt et al., 2010, 2011; Spunt and Lieberman, 2012). Second, contrary to our findings, there was significantly stronger dmPFC activation in the exemplar (low construal level) than in the category task (high construal level) for objects, although the inverse was true for the why (high construal level) vs. how (low construal level) task for persons. At present, we do not have a satisfactory explanation for these discrepant findings.

### Impact of constraint

As previously outlined, we hypothesized that the activation of the dmPFC in high compared to low construal (Baetens et al., 2014) might have been driven by the less constrained nature of the high construal conditions (i.e., they provide less of a clear-cut guiding strategy and rely more heavily on self-guided processes). Based on this hypothesis, one might have expected stronger dmPFC activation in low compared to high constraint in the present study. Yet, we found no differences in dmPFC activation between different levels of constraint. (Note that this holds not only for the conjunction analysis, but for the individual conditions as well, see Table S2). The comparison of low vs. high constraint didn't yield any significant activation differences, in the dmPFC or elsewhere. Only the supplementary analysis, focusing on the onset of the trial, did produce significant activation, in the posterior cingulate cortex/retrosplenial cortex (BA30, see Table S1)—previous investigations have implicated this region in unconstrained (vs. constrained) processes before (Schubert et al., 1999; Mason et al., 2007b; Soon et al., 2008). Conversely, high constraint conditions were associated with greater occipital activations (BA 17, 18) than low constraint conditions. Given the involvement of these regions in horizontal saccade movements (Darby et al., 1996), we believe these activations most likely reflect the requirement to take two visual stimuli into account at the same time in these conditions.

While we did not find the predicted impact of constraint on dmPFC activation, it would be premature to rule out this alternative account based on the present results. Specifically, it is quite possible that our manipulation of constraint failed because it was too subtle compared to previous studies, which pitted totally free choices against fully determined rule-governed decisions (e.g., Rowe et al., 2008). Further, it is conceivable that the task to generate visual or categorical *matches* did not so much constrain the generation process itself, but rather the selection process amongst generated responses. That is, participants may have generated categories or descriptions that applied to one of the pictures under all conditions, but only applied a more constrained selection process in matching these categories or descriptions to the two pictures in the constrained condition.

### Limitations

Several limitations of the present study must be addressed. First, this study is prone to limited experimental control. Participants were asked to engage in different cognitive processes, but as they responded only covertly, it is difficult to verify their performance. Nevertheless, manipulation checks—the verification probes (i.e., questions after each trial about possible categories or visual features that participants had been thinking of)—seem to suggest that participants complied with the instructions. Participants practically always correctly rejected implausible probes, and the plausible probes in more difficult trials (i.e., high constraint conditions) resulted in greater acceptance of the experimenter-provided answers. Further, participants who found the task difficult, generated plausible probes less often by themselves. Nevertheless, a crucial challenge for future research is to create experimental designs enabling the study of high-level construal processes, while at the same time achieving better experimental control.

Second, differences in activation between construal level conditions could be due to task difficulty. Specifically, as participants had to generate responses during 7 s, perhaps they spent a larger fraction of this period idly in the (more difficult) high construal level conditions, because they couldn't think of any further responses. The greater dmPFC involvement in the high construal level conditions could then reflect greater *default network* activation (Gusnard et al., 2001). This network encompasses regions often showing relatively high metabolic activity in the absence of a specific task-directed context and revealing a high overlap with brain areas involved in social mentalizing (Andrews-Hanna et al., 2010). This account seems unlikely given that a parametric analysis did not show any relation between difficulty and dmPFC activation. Moreover, there was no dmPFC activation in the comparison of high and low constraint. As the number of possible correct responses was strictly smaller in the high constraint conditions (as reflected in the subjective difficulty ratings), it seems therefore highly improbable that task difficulty differences underlie the dmPFC involvement in high vs. low construal.

Lastly, as noted before, it is very well possible that our failure to find any impact of constraint on dmPFC activation was due to the more subtle nature of our constraint manipulation compared to other studies. To gain accurate understanding of the relation between task constraint and dmPFC activation, it will be necessary to investigate a wider range of levels and implementations of constraint in the future.

### Conclusion

In the present study, we replicated earlier findings of stronger dmPFC activation during high construal (generating categories) than low construal (visual descriptions) of objects. We found no impact whatsoever of the degree of constraint on activation of the dmPFC. However, further research is needed to rule out the possibility that this dmPFC involvement is due to lower constraints inherent to the generation of categories (as opposed to the description of visual properties).

## Author contributions

Conception and design of the study: KB, NM, FV. Acquisition of the data: KB, NM. Analysis and interpretation of the data: KB, NM, FV. Drafting/revising the manuscript: KB, NM, FV. Final approval: KB, NM, FV.

## Funding

This research was funded by a Ph.D. fellowship of the Research Foundation—Flanders (FWO).

### Conflict of interest statement

The authors declare that the research was conducted in the absence of any commercial or financial relationships that could be construed as a potential conflict of interest.
