# Supplementary material for: The Dorsal Medial Prefrontal Cortex Is Recruited by High Construal of Non-social Stimuli
Source: Front Behav Neurosci. 2017 Mar 14;11:44. doi: 10.3389/fnbeh.2017.00044 (PMC5349082; doi:10.3389/fnbeh.2017.00044)
Supplement: Supplementary file 1 [file Table1.DOCX]

| **Table S1.** Results of conjunction analyses (versus null), all at threshold *p* <.05 (FWE-corrected, number of voxels ≥ 10), trial duration 0 s. | | | | | | | | | | |
| --- | --- | --- | --- | --- | --- | --- | --- | --- | --- | --- |
|  |  |  |  | MNI coordinates | | |  |  |  |  |
| *Contrast* | *Anatomical region* | *BA* |  | *x* | *y* | *z* | *t* |  | *k* |  |
| High Construal > Low Construal | Dorsomedial Prefrontal Cortex | 8 |  | -12 | 50 | 40 | 5.46 |  | 1123 |  |
| *(for both levels of Constraint)* | **Middle Temporal Gyrus** | 21 |  | -52 | -4 | -26 | 5.39 |  | 518 |  |
|  | Posterior Cingulate | 29 |  | -14 | -50 | 4 | 7.05 | ^**^ | 2874 | _a_ |
|  |  | 31 |  | -6 | -54 | 18 | 7.56 | ^**^ | 2874 | _a_ |
|  | Angular Gyrus | 39 |  | -42 | -62 | 28 | 6.25 | ^*^ | 1487 | _b_ |
|  | **Precuneus (inferior)** | 31 |  | -2 | -64 | 24 | 7.37 | ^**^ | 2874 | _a_ |
|  | Inferior Parietal Lobule | 7 |  | -42 | -74 | 44 | 5.38 |  | 1487 | _b_ |
|  |  |  |  |  |  |  |  |  |  |  |
|  | *Ventromedial Prefrontal Cortex* | *11* |  | *-6* | *36* | *-20* | *5.44* |  |  |  |
|  | *Superior Frontal Gyrus* | *8* |  | *-30* | *16* | *56* | *5.95* | *^*^* |  |  |
|  | *Precentral Gyrus* | *6* |  | *-52* | *-4* | *-26* | *5.35* |  |  |  |
|  | *Posterior Cingulate* | *29* |  | *16* | *-46* | *8* | *6.77* | *^**^* |  |  |
|  | *Cerebellum* |  |  | *16* | *-86* | *-36* | *5.31* |  |  |  |
|  |  |  |  |  |  |  |  |  |  |  |
| Low Construal > High Construal | Left Frontal Cortex | 6 |  | -22 | 0 | 58 | 5.95 | ^*^ | 470 |  |
| *(for both levels of Constraint)* | Precuneus (superior) | 7 |  | 24 | -60 | 56 | 7.56 | ^**^ | 1777 | _c_ |
|  |  | 7 |  | -18 | -66 | 50 | 6.07 | ^*^ | 1486 | _d_ |
|  |  | 7 |  | -24 | -66 | 36 | 5.84 | ^*^ | 1486 | _d_ |
|  | Superior Parietal Lobule | 7 |  | -18 | -62 | 60 | 6.39 | ^*^ | 1486 | _d_ |
|  |  | 7 |  | 26 | -66 | 42 | 5.97 | ^*^ | 1777 | _c_ |
|  |  |  |  |  |  |  |  |  |  |  |
|  | *Inferior Frontal Gyrus* | *44* |  | *50* | *10* | *22* | *5.38* |  |  |  |
|  |  | *9* |  | *48* | *8* | *30* | *5.64* |  |  |  |
|  |  | *9* |  | *-50* | *6* | *28* | *5.87* | *^*^* |  |  |
|  | *Middle Frontal Gyrus* | *6* |  | *26* | *2* | *56* | *5.80* | *^*^* |  |  |
|  | *Inferior Parietal Lobule* | *40* |  | *42* | *-36* | *46* | *5.82* | *^*^* |  |  |
|  |  |  |  |  |  |  |  |  |  |  |
| Low Constraint > High Constraint | **Posterior Cingulate** | 30 |  | -2 | -40 | 20 | 5.57 |  | 213 |  |
| *(for both levels of Construal)* |  |  |  |  |  |  |  |  |  |  |
|  |  |  |  |  |  |  |  |  |  |  |
| High Constraint > Low Constraint | Lingual Gyrus | 18 |  | -8 | -74 | -2 | 10.93 | ^**^ | 13590 | _e_ |
| *(for both levels of Construal)* | Cuneus | 17 |  | 12 | -80 | 10 | 11.64 | ^**^ | 13590 | _e_ |
|  |  | 17 |  | -10 | -82 | 10 | 12.60 | ^**^ | 13590 | _e_ |
|  |  |  |  |  |  |  |  |  |  |  |
|  | *Parahippocampal Gyrus* | *28* |  | *-20* | *-26* | *-8* | *5.29* |  |  |  |
|  |  |  |  |  |  |  |  |  |  |  |
| BA = Brodmannʼs Area; L and R = left and right hemispheres; *t* = t-score at those coordinates (peak value); *k* = cluster size (in voxels). Regions with *k*s that share a subscript originate from the same cluster. Activations in bold are only significant in the model including difficulty as a covariate of no interest; activations in italics are only significant without including difficulty as a covariate of no interest. **p* < .01; ***p* < .001 (FWE-corrected). | | | | | | | | | | |
