# Supplementary material for: The Dorsal Medial Prefrontal Cortex Is Recruited by High Construal of Non-social Stimuli
Source: Front Behav Neurosci. 2017 Mar 14;11:44. doi: 10.3389/fnbeh.2017.00044 (PMC5349082; doi:10.3389/fnbeh.2017.00044)
Supplement: Supplementary file 2 [file Table2.DOCX]

| **S2.** Results of whole-brain analysis, all at threshold *p* <.05 (FWE-corrected, number of voxels ≥ 10), trial duration 7 s. | | | | | | | | | | | |
| --- | --- | --- | --- | --- | --- | --- | --- | --- | --- | --- | --- |
|  |  |  |  | MNI coordinates | | |  |  |  |  |  |
| *Contrast* | *Anatomical region* | *BA* |  | *x* | *y* | *z* | *t* |  | *k* |  |  |
| *High Construal > Low Construal* | Dorsomedial Prefrontal Cortex | 9 |  | -8 | 52 | 38 | 6.81 | ** | 2002 | a |  |
|  |  | 8 |  | -10 | 46 | 46 | 6.54 | ** | 2002 | a |  |
|  | Ventromedial Prefrontal Cortex | 11 |  | -8 | 36 | -16 | 6.98 | ** | 874 |  |  |
|  | Superior Frontal Gyrus | 8 |  | -30 | 16 | 56 | 6.71 | ** | 2002 | a |  |
|  | Middle Temporal Gyrus | 21 |  | -50 | -10 | -22 | 6.42 | * | 673 |  |  |
|  | Posterior Cingulate | 30 |  | -8 | -52 | 16 | 8.92 | ** | 6464 | b |  |
|  | Angular Gyrus | 39 |  | 54 | -66 | 34 | 8.07 | ** | 526 | c |  |
|  |  | 39 |  | 50 | -70 | 40 | 7.38 | ** | 526 | c |  |
|  |  | 39 |  | -48 | -72 | 38 | 8.56 | ** | 6464 | b |  |
|  |  | 39 |  | -42 | -74 | 46 | 7.90 | ** | 6464 | b |  |
|  | Cerebellum |  |  | -6 | -44 | -30 | 5.61 |  | 249 |  |  |
|  |  |  |  | 40 | -74 | -38 | 6.29 | * | 634 | d |  |
|  |  |  |  | 16 | -86 | -36 | 7.00 | ** | 634 | d |  |
|  | *Parahippocampal Gyrus* | *28* |  | *16* | *-14* | *-24* | *5.25* |  |  |  |  |
|  |  | *35* |  | *-22* | *-32* | *-18* | *5.30* |  |  |  |  |
|  | *Cerebellum* |  |  | *-6* | *-50* | *-50* |  |  |  |  |  |
|  |  |  |  |  |  |  |  |  |  |  |  |
| *Low Construal > High Construal* | Inferior Frontal Gyrus | 46 |  | 46 | 40 | 12 | 6.13 | ** | 402 |  |  |
|  |  | 9 |  | 52 | 10 | 32 | 8.62 | ** | 2412 | e |  |
|  |  | 44 |  | 50 | 8 | 20 | 7.97 | ** | 2412 | e |  |
|  |  | 9 |  | -48 | 6 | 28 | 10.72 | ** | 2613 | f |  |
|  | Superior Frontal Gyrus | 6 |  | 26 | 0 | 58 | 8.99 | ** | 2412 | e |  |
|  |  | 6 |  | -22 | -2 | 58 | 10.26 | ** | 2613 | f |  |
|  | Insula | 13 |  | -36 | -2 | 10 | 6.01 | ** | 88 |  |  |
|  | Middle Frontal Gyrus | 6 |  | -28 | -4 | 52 | 9.86 | ** | 2613 | f |  |
|  | Thalamus |  |  | -18 | -28 | 2 | 5.62 |  | 185 |  |  |
|  | Precuneus (superior) | 7 |  | -30 | -48 | 52 | 12.30 | ** | 11807 | g |  |
|  |  | 7 |  | 24 | -60 | 56 | 12.85 | ** | 11866 | h |  |
|  | Inferior Temporal gyrus | 37 |  | 54 | -60 | -8 | 12.14 | ** | 11866 | h |  |
|  | Middle Temporal Gyrus | 37 |  | -46 | -66 | 2 | 12.18 | ** | 11807 | g |  |
|  | Precuneus (superior) | 7 |  | -24 | -68 | 38 | 13.53 | ** | 11807 | g |  |
|  | Superior Parietal Lobule | 7 |  | 26 | -68 | 42 | 13.27 | ** | 11866 | h |  |
|  | Cerebellum |  |  | -22 | -68 | -48 | 5.78 | ** | 94 |  |  |
|  |  |  |  | 16 | -70 | -44 | 6.68 | ** | 36 |  |  |
|  |  |  |  |  |  |  |  |  |  |  |  |
| *Low Constraint > High Constraint* | **Anterior Cingulate** | 32 |  | -10 | 36 | -10 | 5.25 |  | 323 |  |  |
|  | **Parahippocampal Gyrus (posterior)** | 30 |  | 32 | -40 | 4 | 6.52 | ** | 6548 | i |  |
|  | **Insula** | 13 |  | -24 | -42 | 10 | 6.04 | * | 6548 | i |  |
|  |  | 13 |  | 28 | -44 | 24 | 5.54 |  | 6548 | i |  |
|  | *Corpus Callosum* |  |  | *16* | *30* | *6* | *5.43* |  |  |  |  |
|  | *Caudate Nucleus* |  |  | *20* | *16* | *16* | *5.28* |  |  |  |  |
|  | *Sub-Gyral* |  |  | *-20* | *-44* | *12* | *5.86* | *** |  |  |  |
|  |  |  |  |  |  |  |  |  |  |  |  |
| *High Constraint > Low Constraint* | Middle Frontal Gyrus | 10 |  | -48 | 48 | -4 | 5.68 |  | 627 |  |  |
|  | Middle Frontal Gyrus | 9 |  | 50 | 32 | 32 | 5.92 | * | 1814 | j |  |
|  |  | 9 |  | 48 | 26 | 38 | 6.40 | *** | 1814 | j |  |
|  | Posterior Medial Frontal Cortex | 8 |  | 6 | 26 | 48 | 5.86 | * | 705 |  |  |
|  | Inferior Frontal Gyrus | 47 |  | 32 | 26 | -10 | 5.28 |  | 261 |  |  |
|  | **Parahippocampal Gyrus (anterior)** | 28 |  | 22 | -26 | -6 | 6.33 | *** | 181 |  |  |
|  | **Inferior Parietal Lobule** | 40 |  | 34 | -48 | 50 | 5.26 |  | 770 |  |  |
|  | **Posterior Cingulate** | 30 |  | -10 | -72 | 12 | 14.83 | ** | 23572 | k |  |
|  | Lingual Gyrus | 18 |  | -10 | -76 | -2 | 15.68 | ** | 23572 | k |  |
|  | Cuneus | 17 |  | 12 | -80 | 6 | 15.60 | ** | 23572 | k |  |
|  | *Middle Frontal Gyrus* | *47* |  | *-42* | *42* | *-10* | *6.26* | * |  |  |  |
|  |  | *9* |  | *-50* | *18* | *40* | *5.96* | * |  |  |  |
|  | *Precuneus (superior)* | *7* |  | *-28* | *-50* | *50* | *5.40* |  |  |  |  |
|  | *Cuneus* | *18* |  | *-8* | *-88* | *8* | 17.14 | ** |  |  |  |
| *Interaction: (High Construal, Low Constraint > High Construal, High Constraint) > (Low Construal, Low Constraint > Low Construal, High Constraint)* | | | | | | | | | | | |
|  | No significant clusters. |  |  |  |  |  |  |  |  |  |  |
|  |  |  |  |  |  |  |  |  |  |  |  |
| *Interaction: (Low Construal, Low Constraint > Low Construal, High Constraint) > (High Construal, Low Constraint > High Construal, High Constraint)* | | | | | | | | | | | |
|  | *Middle Occipital Gyrus* | *19* |  | *34* | *-84* | *24* | *5.58* |  |  |  |  |
|  | *Parahippocampal Gyrus* | *37* |  | *28* | *-38* | *-14* | *5.50* |  |  |  |  |
| BA = Brodmannʼs Area; *t* = t-score at those coordinates (peak value); *k* = cluster size (in voxels). Regions with *k*s that share a subscript originate from the same cluster. Activations in bold are only significant in the model including difficulty as a covariate of no interest; activations in italics are only significant without including difficulty as a covariate of no interest. **p* < .01; ***p* < .001 (FWE-corrected). | | | | | | | | | | | |
